# Risk factors and prevalence of Human Immunodeficiency Virus and Syphilis, among prisoners in Duhok city, Kurdistan Region, Iraq

**DOI:** 10.1186/s12879-025-11240-7

**Published:** 2025-07-01

**Authors:** Masood Abdulkareem Abdulrahman, Farhad Ismail Shahab, Ibrahim Mohammed Abdullah, Arazoo Issa Tahir, Bland Bayar Khaleel, Nazik Abdulraheem Abdulkarim

**Affiliations:** 1 Public Health Department, College of Health and Medical Technology, Shekhan/ Duhok Polytechnic University, 61 Zakho Road, Mazi Qr, Duhok Governorate, Kurdistan Region, Duhok, Shekhan 1006 Iraq; 2 Medical Laboratory Technology Department, College of Health and Medical Technology, Shekhan/ Duhok Polytechnic University, Duhok Governorate, Kurdistan Region, 61 Zakho Road, Mazi Qr, Duhok, 1006 Iraq

**Keywords:** Syphilis, HIV, Prisons, Duhok City

## Abstract

**Background:**

It is well recognized that jails are high-risk settings for the transmission of Sexually Transmitted Infections (STIs) like HIV and Syphilis. This study was done to detect the prevalence and risk factors of HIV and Syphilis among inmates.

**Methods:**

A cross-sectional survey was carried out among inmates in two main jails inside Duhok City. A total of 1033 inmates were investigated between January and February 2024. Participants filled out a personal risk factor questionnaire. Serum samples were tested for HIV1 + 2 and syphilis antibodies using a qualitative ELISA method.

**Results:**

The average age of the participants was 35.9 ± 10.62 years, with a range of 13–82 years. Out of the 1013 prisoners, 963 (95.1%) were men. Ever married inmates constituted (66.34)%, and (56.1%) of the prisoners were urban dwellers. Regarding their nationality, almost all of the participants were Iraqis (97.0%). It was reported that 68.11% of individuals were self-employed, and 62.8% of them were smokers.Only six cases of Syphilis were detected among male inmates. So, the prevalence rate was 0.59%. Four cases were above 51 years. Age groups were significantly (*P* =.001) associated with Syphilis cases, while there was no significant statistical association with other demographic characteristics (sex *P* =.850, residency *P* =.990, nationality = 0.165, extramarital status *P* =.187, occupation *P* =.261), and there was no significant statistical association between infected intimates with all studied risk factors (drug abuse *P* =.990, homosexuality *P* =.470, extramarital sex *P* =.118, blood transfusion *P* =.990, surgical operations *P* =.425, tattooing *P* =.990. The prevalence of HIV was zero.

**Conclusions:**

Inmates in Duhok City had a low prevalence rate of syphilis and no HIV infections were recorded. Since inmates are a vulnerable group that frequently enters and exits the general population, health data on this population is crucial.

**Supplementary Information:**

The online version contains supplementary material available at 10.1186/s12879-025-11240-7.

## Background

Syphilis continues to pose a significant threat to public health and a challenge to global health systems. It is defined as a chronically progressing infection brought on by the bacteria *Treponema pallidum*, which is mostly spread by blood, vertical transmission, and sex (oral, vaginal, and anal). Half of infections are asymptomatic, which makes controlling the transmission chain difficult, particularly if treatment is insufficient [1]. The World Health Organization (WHO) estimates that 8 million adults between 15 and 49 years old acquired syphilis in 2022 [2].

Although more than 30 infections are known to be sexually transmitted, only 8 were linked to a high incidence of morbidity and mortality, including syphilis and HIV [3].

Syphilis is more common in prisoners than in the general population, with a systematic study reporting a frequency of 2.89%. Because incarceration disrupts people’s social and sexual networks, it may lead to the establishment of high-risk sexual relationships.

Additionally, the breakdown of social networks and support systems that occurs during incarceration raises the possibility that risk factors for STIs (such as drug abuse, depression, or anxiety, and an increase in the number of sexual partners) will manifest, as well as the possibility that they will be acquired or transmitted to the community in the future [4, 5]. Venereal syphilis is uncommon in Iraq and other countries in the region. Moreover, little data is available on the prevalence of this infection in Iraq. As information about STIs in Islamic countries is limited, data on the prevalence and epidemiology of syphilis in Iraq are either limited or not accurate. According to the latest WHO data published in 2017, syphilis deaths in Iraq reached to 166 or 0.09% of the total death [6].

In prisons, high-risk transmission behaviours (such as continued drug injection and syringe sharing, risky sexual conduct, tattooing, and piercing) may result in rapid and severe disease transmission and development [7]. In addition, the influence of socioeconomic aspects, such as low education, low income, and difficult access to health services, are also determining factors for maintenance and emergence of infection [8].

HIV remains a major global public health issue (about 39.9 million people living with HIV at the end of 2023) [9]. Transmission continues in all countries throughout the world. The estimated global HIV prevalence in prisoners is 3% [10]. Drug use, needle sharing, tattooing using improvised and unsterile equipment, high-risk sex, and rape make prisons a high-risk setting for HIV transmission. The immune system is weakened by violence, drug abuse, stress, starvation, and overcrowding, which increases the risk of illness for those living with HIV.

Most people in prisons around the world are men between the ages of 19 and 35, and many of them are jailed for drug-related offenses. Before they are incarcerated, young men who are drug abusers are already more likely to contract HIV [11]. In a systematic review conducted for 72 studies documenting the prevalence of HIV, 2,275,930 adult male and female inmates were included in the many studies, the pooled estimate of HIV prevalence was 3.4% و and the prevalence of HIV varied significantly between different studies (from zero in Bosnia and Herzegovina to more than 20% in Iran, Zambia, and Spain [12]. The Joint United Nations Program on HIV/AIDS (UNAIDS) has revealed that around 270,000 people are living with HIV in the Middle East and North Africa (MENA) region, an overall HIV prevalence of 0.1% among adults aged 15 to 49, and the prevalence of HIV in Iraq was less than 0.1% that one of the lowest rates in the world.The low prevalence of HIV in general populations of MENA has been attributed in part to the region’s religious (Islamic) and cultural norms, which discourage premarital sex, encourage faithfulness within marriage, and adopting the practice of male circumcision [13]. Kurdistan Region of Iraq (KRI) has one of the lowest global HIV rates, attributed to adherence to social, cultural, and religious values, public health programs. Out of over 307,000 tests conducted in 2023, at least 44 HIV cases have been detected, of whom 28 are foreign nationals while the rest are Iraqi citizens. HIV tests are mandatory in the KR for obtaining residency permits, and those who had positive results are deported [14].

In Iraq, a few studies are conducted on HIV due to political instability since the first Gulf war which followed by second Gulf war and UN sanctions on Iraq for about seven years. There is no nationwide sero-surveillance data system related to HIV. The present screening program focused on some highly risk groups in the community like premarital, foreigners (workers and visitors), blood donors, active TB cases, and antenatal care but did not include the inmates up to the present time [15]. In Duhok city, there are health care centers inside all jails (correctional centers) which provide primary health care services, and the suspected cases of syphilis or HIV or other communicable diseases like HBV or HCV are referred to the main Teaching Azadi hospital inside Duhok city for the required management.

As a matter of fact, one of the neglected groups of individuals who are at risk for acquiring the infection are prisoners, and it is well recognized that jails are high-risk settings for the transmission of STIs like HIV and Syphilis, and Individuals infected with HIV are eight times more likely to become infected with syphilis than the general population [16]. Thus, the aim of this study is to detect the prevalence and risk factors of HIV and syphilis among prisoners in Duhok jails. To the best of our knowledge, it is the first study in this field to be done in the KRI.

## Methods

### Study design and setting

In Duhok City, a cross-sectional survey was carried out in January 2024. There are two main jails inside Duhok City: the main jail for adult males called Zerka Jail and the other for adolescents and female adults called Etiti jail.

### Study population

The study population is made up of all available prisoners in the two jails at the time of the study. At Zerka Jail, 945 prisoners were included, and at Etiti Jail, 50 adult females and 18 adolescent prisoners were included.

#### Exclusion criteria

1. Any mentally unstable inmate or one who had psychiatric disorders.
2. Inmates who were very sick and/or were hospitalized.

### Data collection

A face-to-face interview technique was used for data collection. The data was collected by well-trained staff through using a validated questionnaire. There are two sections in the questionnaire (see attached file). The questionnaire was developed by the researchers. It was validated by a panel of professionals who contributed some notes and changes. Their notes were considered by the researchers and appropriate modifications were made accordingly.

The first part includes demographic characteristics (nationality, age, marital status, address of residence, and family history). The second part was related to risk factors (tattooing, number of sexual partners, sharing contaminated materials such as toothbrushes and shaving blades, blood transfusion history, drug abuse, and dental extraction).

### Laboratory investigations

A gel tube was used to collect 5 milliliters of venous blood from each participant for testing of HIV1 + 2 antibodies and anti-syphilis. Cool boxes were employed to transfer the samples from the prisoners to Duhok Central Laboratory to maintain the integrity of the samples. The blood was centrifuged at 2,500 rpm for 10 min, after which the supernatant (serum) was collected for analysis. The serum samples were tested for HIV1 + 2 and syphilis IgG antibodies using a qualitative ELISA with the Biorex Diagnostics kit (UK), following the manufacturer’s recommendations. The results were read through employ a microplate reader to measure the absorbance at 450 nm (reference filter: 620–650 nm), the cutoff value was calculated according to the manufacturer’s instructions (usually by using the mean absorbance of the negative control plus a specific factor). Interpretation of results into three categories:

- Positive Result: Absorbance above the cutoff value indicates the presence of syphilis antibodies.
- Negative Result: Absorbance below the cutoff value indicates the absence of detectable antibodies.
- Equivocal Result: If the absorbance is near the cutoff, repeat the test or confirm with additional testing.

### Ethical considerations

Informed consent to participate was obtained from all participants. All information related to demographic and risk behavior including extramarital sex, homosexuality, and drug abuse were dealt with confidentiality. The interview and blood sample collection were done at the prison health center by two trained health staff of the same sex of the inmates.

### Statistical analysis

The data was entered and analyzed using SPSS software version 24. Descriptive statistics and chi-square tests were used. The Fisher’s exact test was used when more than 20% of table’s cells had an expected value of less than 5. A P value ≤ 0.05 was considered significant.

## Results

The average age of the participants was 35.9 ± 10.62 years, with a range of 13–82 years, of the 1013 prisoners, 95.1% were men. The percentage of married inmates was 61.6%. 56.1% of the prisoners were urban dwellers. Furthermore, almost all participants were Iraqis (97%). In addition, 42.8% of individuals were wage workers, and 62.8% of people were smokers (Table 1).

**Table 1:** Sociodemographic characteristics of the study population (*n* = 1013)

**Variables**	**No.**	**%**
Mean age, years	35.9±10.62 years, with a range of 13–82 years	
Sex		
Male	963	95.1
Female	50	4.9
Marital status		
Single	341	32.66
Ever married	672	66.34
Residency		
Rural	138	13.6
Urban	568	56.1
Semi-urban	307	30.3
Nationality		
Iraqi	983	97.0
Others	16	3.0
Occupation		
Public employee	198	19.55
Private employee	690	68.11
Housewife	39	3.85
Student	86	8.49
Smoking status		
Smokers	637	62.8
Non-smokers	376	37.2

Only six cases of syphilis were detected among male inmates, so the prevalence was 0.59%. Four cases were above 51 years, most of them (5 cases) were residents of urban areas. Five of the cases were Iraqis, and one of them was Syrian. All cases were married individuals. Regarding the occupation, two cases were public employees, two were private sector employees while one of them was retired, and the other was wage employee. There was a significant statistical association between cases of syphilis and age groups mainly ≥ 51 years age group (*P* =.001), while there was no significant statistical association with other demographic characteristics (Sex *P* =.850, Residency *P* =.990, Nationality = 0.165, Marital status *P* =.187, Occupation *P* =.261) (Table 2).

**Table 2:** Prevalence of syphilis by sociodemographic characteristics.

**Variables**		Total	Syphilis 6/1013		*P*-Value
			Positive	Negative	
Sex	Male	963	6 (0.62)	957 (99.38)	0.850
	Female	50	0 (0.0)	50 (100)	
Age group in years	13-30	344	0 (0.0)	344 (100.0)	0.001*
	31-50	571	2 (0.4)	569 (99.6)	
	51 and above	98	4 (4.1)	94 (95.9)	
Residency	Rural	138	1 (0.72)	137 (99.28)	0.990*
	Urban	875	5 (0.57)	870 (99.43)	
Nationality	Iraqi	983	5 (0.5)	978 (99.5)	0.165*
	Others	30	1 (3.3)	29 (96.7)	
Marital status	Single	341	0 (0.0)	341 (100)	0.187*
	Ever married	672	6 (0.89)	666 (99.11)	
Occupation	Public employee	198	3 ( 1.5)	190 (98.5)	0.261*
	Private employee	690	3 ( 0.4)	687 (99.6)	
	Housewife	39	0(0.0)	39(100.0)	
	Student	86	0(0.0)	86 (100.0)	

*Fisher’s exact test was used

All syphilis cases had a negative history of drug abuse. One case had a history of homosexuality, and another had a history of blood transfusion. Three cases had a history of extramarital sex. The other three cases had a history of previous surgical operations. Two cases had a history of tattooing, and all cases had a history of tooth extraction. There is no significant statistical association between infected intimates with all studied risk factors (drug abuse *P* =.990, homosexuality *P* =.470, extra marital sex *P* =.118, blood transfusion *P* =.990, surgical operations *P* =.425, tattooing *P* =.990, and tooth extraction *P* =.088) (Table 3).

**Table 3:** Prevalence of Syphilis among intimates in relation to risk factors (*n*=1013)

**Risk Factors **		**Total**	Syphilis		*P*-Value
			Positive	Negative	
Drug abuse	Yes	127	0 (0.0)	127 (100)	0.990*
	No	886	6 (0.68)	880 (99.32)	
Homosexuality	Yes	87	1 (1.14)	86 (98.8)	0.470*
	No	926	5 (0.54)	921 (99.46)	
Sex outside family	Yes	218	3 (1.37)	215 (98.6)	0.118*
	No	795	3 (0.38)	792 (99.62)	
Blood transfusion	Yes	164	1 (0.6)	163 (99.4)	0.990*
	No	849	5 (0.59)	844 (99.41)	
Surgery	Yes	353	3 (0.84)	350 (99.16)	0.425
	No	660	3 (0.46)	657 (99.54)	
Tattooing	Yes	338	2 (0.6)	336 (99.4)	0.990*
	No	675	4 (0.6)	671 (99.4)	
Tooth extraction	Yes	623	6 (0.97)	617 (99.03)	0.088*
	No	390	0 (0.0)	390 (100)	

*Fisher’s exact test was used

The routes of drug administration among drug users arranged as oral route (41.7%), inhalation (38.6%), injection (4.7%), and multiple route (15.0%). The subcutaneous route of injectable drug administration is more common (60%) than the intramuscular route (40%). Those inmates who used injectable route of drug, 44.4% of them shared it with others. There were significant statistical association between sex with drug addiction (*P* =.001) and routes of drug administration (*P* =.039). On the other hand, there was insignificant statistical association with routes of injection (*P* =.999), and sharing of injections *P* =.069) (Table 4).

**Table 4:** Routes of drug administration among drug abusers

Variable	Options	Sex			*P*. value
		Male No. (%)	Female No. (%)	Total No. (%)	
Drug abuse	Yes	113 (11.7)	14 (28.0)	127 (12.5)	0.001
	NO	850(88.3)	36(72.0)	886 (87.5)	
Routes of drug administration (*n* = 127)	Injections	4 (3.1)	2 (1.6)	6 (4.7)	0.039 *
	Oral	44 (34.6)	9 (7.1)	53 (41.7)	
	Inhalation	47 (37.0)	2 (1.6)	49 (38.6)	
	More than one route	18 (14.2)	1 (0.8)	18 (15.0)	
Routes of Injection (*n* = 15)	Intravenous (IV)	5 (33.3)	1 (6.7)	6 (40.0)	0.999 *
	Subcutaneous (SC)	7 (46.7)	2(13.3)	9 (60.0)	
Sharing of injections	Yes	5 (27.8)	3 (16.7)	8 (44.4)	0.069 *
	NO	10(55.6)	0 (0.0)	10 (55.6)	

*Fisher’s exact test was used

No HIV cases were detected among inmates.

## Discussion

Prisons are frequently favorable environment for the spread of HIV, and syphilis [17]. The number of female and adolescent inmates in the study sample is near to the percentages of two categories in the Iraqi’s jails female (2.6%) adolescents (3.6%) [18]. Studies conducted in Iraq among various populations, including those in KRI, revealed a low frequency of syphilis. However, based on our research on the literature review, none have been published on prisoners. In the present study, out of 1013 participants, 6 cases were positive with a percentage of (0.59). This may be approximate to the prevalence of syphilis among blood donors in three provinces of KRI in the time period between 2013 and 2015. In Erbil province, 164177 donors were tested, 82370 in Sulimany, and 69987 in Duhok. All were tested for HIV and Syphilis. In Erbil, the prevalence of syphilis was 0.21%, and only one HIV case was detected. While in Sulimanya, there were 2 HIV cases 2 and the prevalence rate of syphilis was 0.353%. But in Duhok province, the prevalence of HIV was zero and syphilis was 0.342% [19]. In another study done in Basrah province in the southern part of Iraq, the prevalence of syphilis among 197 898 samples of blood donors was 0.36% in 2021 [20]. In comparison with other studies conducted in jails worldwide, Nafees et al. found that among 4915 jail inmates (4498 male and 417 females) in two jails of Lahore, Pakistan, the total HIV prevalence rate was 2.01% [21], Kazi et al. found that among 365 male inmates in a jail in Karachi 2% infected with HIV, and 8.9% had syphilis [22]. Niode et al. found that 1.1% of studied inmates at the Correctional Institution in Manado, Indonesia were infected with HIV, and 12.8% tested positive for syphilis []. In a study done for 662 male inmates of the Casa Circondariale-Genova Marassi,, Italy, the prevalence of HIV was 1.8% and for syphilis was 1.3% [23]. Another study done by Ravlija et al. in Bosnia and Herzegovina for 620 inmates in 10 jails, the prevalence of syphilis was 0.5%, and no any case of HIV was detected, these rates are close to our study results [24] The prevalence of HIV infection among 5,530 inmates recruited for a statewide bio-behavioral study in Iran that was carried out in 27 prisons was 2.1%. HIV prevalence among drug users was assessed in three Iranian studies: 15.1% among 252 inmates in Bandar Abbas and Roodan, 5.8% among 121 inmates in Gorgan, and 6.4% among 970 inmates in Isfahan [25].

In our study, most of the cases were in the age group above 51 years. While in an Indonesian study, most cases were of younger age (25–49 years) [26]. In the current study, there was no significant association between syphilis infection with many factors (surgical operations, blood transfusion, marital status, extramarital sex, and drug abuse). Same results were found by Kazi et al. [22].

Although all syphilis cases had a history of tooth extraction, other risk factors displayed no significant statistical impact. One of the most concerning risk factors in a Brazilian research on female inmates was the majority of participants’ irregular condom usage [27]. This reckless behavior may be the primary cause of the disease’s rising occurrence. Educational initiatives must be carried out to lower the incidence and danger of STIs [28]. Our findings indicate that the prevalence of syphilis does not significantly vary across research groups and it is unaffected by homosexuality or extramarital sex.

This might be a result of the low rate of extramarital partnerships among Iraqis, which lowers the risk of STIs [29]. In another study, the elevated rate of risky sexual practices, such as unprotected sexual contact and sex with numerous partners, which are typical in prison settings, is one of the main variables contributing to the findings in this study [30]. Furthermore, drug abuse and the sharing of injectable supplies within jails might potentially be factors contributing to syphilis outbreak and other blood-borne infections [7]. On the contrary, Jiang stated that intravenous drug abuse, particularly the sharing of injection equipment such as needles and syringes, can increase the risk of blood-borne infections such as syphilis [31]. Since almost all prisoners are eventually released, the potential for continuous transmission of STIs in the free society has significant implications for public health. Mathematical models suggests that half of all HIV new cases among people who inject drugs in Eastern Europe and Central Asia could be attributed to a history of incarceration. Therefore, identification and early treatment of infected inmates is fundamental not only to limit the disease burden and costs within the prison but also to reduce the risk of STI transmission to the general population upon the release from prisons. Besides, prisoners are not a static population and often move back and forth to the outside world and could circulate the infection. In fact, prisons provide various conditions for both the spread and prevention of STIs [32]. We should understand the true scale of this public health problem, and to develop new effective strategies for control and prevention, we need; prison-related risk factor identification, and more reliable prisons STI incidence and prevalence rates compared to the general population.

### Study limitations

The study has few limitations that undermined its ability to get accurate information from participants on past sexual behavior, homosexuality, extramarital sex, and drug abuse due to personal or social pressures.

## Conclusions

Inmates in Duhok City had a prevalence rate of syphilis and HIV that is comparable to the overall community. Because inmates are a vulnerable group that frequently enters and exits the general population, health data on this population is crucial. The prevalence of sexual activity in prisons is unclear and is thought to be much underreported, and inmate screening is not a regular practice. Targeting high-risk groups is therefore essential to lower the incidence of STIs. All inmates should be offered the opportunity to undergo a comprehensive screening for all STIs, including those that are currently neglected. We recommend an ongoing continuous surveillance of inmates for common STIs.

## Supplementary Information

Supplementary Material 1.

## Data Availability

The data is available on request, all data can be requested from the corresponding author through this email: masood.abdulkareem@dpu.edu.krd.

## Abbreviations

HIV: Immunodeficiency Virus
WHO: World Health Organization
STIs: Sexually Transmitted Infections
ELISA: Enzyme-linked immunosorbent assay
SPSS: Statistical Package for the Social Sciences
KR: Kurdistan Region
