# Supplementary material for: Risk factors and prevalence of Human Immunodeficiency Virus and Syphilis, among prisoners in Duhok city, Kurdistan Region, Iraq
Source: BMC Infect Dis. 2025 Jul 1;25:869. doi: 10.1186/s12879-025-11240-7 (PMC12219918; doi:10.1186/s12879-025-11240-7)
Supplement: Supplementary file 1 — Supplementary Material 1. [file 12879_2025_11240_MOESM1_ESM.pdf]

Risk factors and prevalence of Human Immunodeficiency Virus and Syphilis, among prisoners in Duhok city, Kurdistan Region, Iraq

Questionnaire

Person name: ID:

Age: years

Sex: Male ☐ Female ☐

Prisoner ☐ Prison administrating staff ☐

Nationality: Iraqi ☐ Syrian ☐ Turkish ☐ Iranian ☐ other specify: ☐

Residency: Urban ☐ Semi Urban ☐ Rural ☐

Occupation: Public Employer ☐ Private Employer ☐ Retired ☐ Student ☐
Wage Employer ☐ Vacation Employer ☐ Others specify…………..

History of:

Blood Transfusion ☐ Surgery ☐ Tattooing ☐ Tooth Extraction ☐

Ask inside prison about Sharing: Toothbrush ☐ Shaving ☐

Drug Abuse: Yes ☐ NO ☐

If Yes Type Injection ☐ Oral ☐ Inhalation ☐

If Injection Route IM ☐ SUB ☐

Are you sharing injections Yes ☐ No ☐

Sexual Behavior

Homosexual	Yes	☐	No	☐
Outside of Family	Yes	☐	No	☐

Are you smoker? Yes ☐ No ☐

How many cigarettes do you smoke per day

1- 5 6- 10 11- 20 21- 30 31- 40 41- 50 and more

Marital Status: Single ☐ Married ☐ Widowed ☐ Divorced ☐

HIV Test	Positive	☐	Negative	☐
TPHA Test	Positive	☐	Negative	☐.
